# Contribution of Microorganisms to Biogenic Amine Accumulation during Fish Sauce Fermentation and Screening of Novel Starters

**DOI:** 10.3390/foods10112572

**Published:** 2021-10-25

**Authors:** Xinxiu Ma, Jingran Bi, Xinyu Li, Gongliang Zhang, Hongshun Hao, Hongman Hou

**Affiliations:** 1 School of Food Science and Technology, Dalian Polytechnic University, No. 1, Qinggongyuan, Ganjingzi District, Dalian 116034, China; maxinxiu@hotmail.com (X.M.); bijr@dlpu.edu.cn (J.B.); lixinyu990519@163.com (X.L.); zgl_mp@163.com (G.Z.); beike1952@163.com (H.H.); 2 Liaoning Key Lab for Aquatic Processing Quality and Safety, No. 1, Qinggongyuan, Ganjingzi District, Dalian 116034, China

**Keywords:** fish sauce, biogenic amines, microbial community dynamics, starter, correlation analysis

## Abstract

In this study, high-throughput sequencing and culture-dependent and HPLC methods were used to investigate the contribution and regulation of biogenic amines (BAs) by dominant microorganisms during fish sauce fermentation. The results showed that the microbial composition constantly changed with the fermentation of fish sauce. *Tetragenococcus* (40.65%), *Lentibacillus* (9.23%), *Vagococcus* (2.20%), *Psychrobacter* (1.80%), *Pseudomonas* (0.98%), *Halomonas* (0.94%) and *Staphylococcus* (0.16%) were the dominant microflora in fish sauce. The content of BAs gradually increased as the fermentation progressed. After 12 months of fermentation, the histamine content (55.59 mg/kg) exceeded the toxic dose recommended by the Food and Drug Administration (FDA). Correlation analysis showed that dominant microorganisms have a great contribution to the accumulation of BAs. By analyzing the BA production capacity of dominant isolates, the accumulation of BAs in fish sauce might be promoted by *Tetragenococcus* and *Halomonas*. Moreover, four strains with high BA reduction ability were screened out of 44 low BA-producing dominant strains, and their influence on BA accumulation in fermented foods was determined. Results demonstrated that *Staphylococcus nepalensis* 5-5 and *Staphylococcus xylosus* JCM 2418 might be the potential starters for BA control. The present study provided a new idea for the control of BAs in fermented foods.

## 1. Introduction

Fish sauce, a seasoning with a unique flavor, is spontaneously fermented from anchovies and salt in a certain proportion, and the fermentation time was more than 6 months [1,2]. Fish sauce, with a particular aroma and rich in amino acids, is often used as a seasoning and nutritional supplement in Southeast Asian countries. In China, fish sauce is mainly popular in eastern coastal areas such as Shandong, Guangdong and Fujian, with an annual output of over 100,000 tons [3,4]. In the process of fish sauce fermentation, a large number of microorganisms are introduced, which may produce biogenic amines from amino acids. Due to increasing occurrences of food-borne disease outbreaks caused by biogenic amines, food safety has received more concern in the production of fish sauce [5].

BAs are small molecule nitrogen-containing compounds which are formed by the decarboxylation or transamination of amino acids by microorganisms [6]. Although BAs have certain physiological functions in the human body, excessive intake may bring safety risks [7]. Due to the hazardous effects of BAs on humans, such as rash, migraine, hypertension and hypotension, the determination of BAs in food products has attracted worldwide attention [5]. Fish products are typical foods containing high contents of BAs [8]. The common BAs in fish sauce are Tryptamine, β-phenylethylamine, putrescine, cadaverine, histamine, tyramine, spermine and spermidine, among which putrescine, cadaverine, histamine and tyramine are the most common BAs [6,9,10]. In particular, some fish sauce products have been reported to contain histamine of 349 to 5487 mg/kg, which exceeded the FDA limit (50 mg/kg) for histamine [11,12,13,14]. Therefore, it is very important to demonstrate the formation mechanism and control of BAs in the fermentation of fish sauce.

The microbial community in naturally fermented fish sauce is complex, but it is a significant factor in determining product quality and safety [15]. Therefore, by defining the succession of bacterial communities and their correlation with BAs, the contribution of microorganisms on the accumulation of BAs in fish sauce fermentation could be explained. Previous studies have shown that only a small number of bacteria may contribute to the formation of biogenic amines [16]. For example, Sang et al. [17] showed that the 30 high BA-producing strains detected in the shrimp paste might be the key factor causing the high BA content in shrimp paste. Microorganisms not only have the ability to produce BAs, but can also reduce BAs by producing amine oxidase [18,19]. Mah et al. [20] found that the content of BAs in Myeolchi-jeot inoculated with *S. xylosus* NO.0538 was 16.0% lower than that in the control group. From this perspective, evaluating bacterial BA production and reduction ability is critical in understanding the BAs accumulation mechanism. Therefore, compared with previous studies, our study not only analyzed the role of microorganisms in BA accumulation, but also identified the strains that can reduce BAs on this basis, which were successfully applied to fish sauce and other fermented foods.

The fish sauce factories in northern China are concentrated in Shandong, and the fish sauce factory that provided samples for us is the largest factory in Shandong, with a complete and representative fermentation process. In this study, the fish sauce samples at different fermentation stages from Rongcheng (Shandong province, PR China) were characterized by BAs assays, high-throughput sequencing analysis of bacterial diversity and cultural isolation of dominant bacteria. The microbial community information was used to clarify the contribution of microorganisms to the accumulation of BAs. Candidate starters isolated from fish sauce to control BA accumulation were screened. This research provides new ideas for improving the quality of fish sauce and reducing the risk of eating fish sauce.

## 2. Materials and Methods

### 2.1. Sample Collection

#### 2.1.1. Fish Sauce Samples Collection

**Figure 1:**
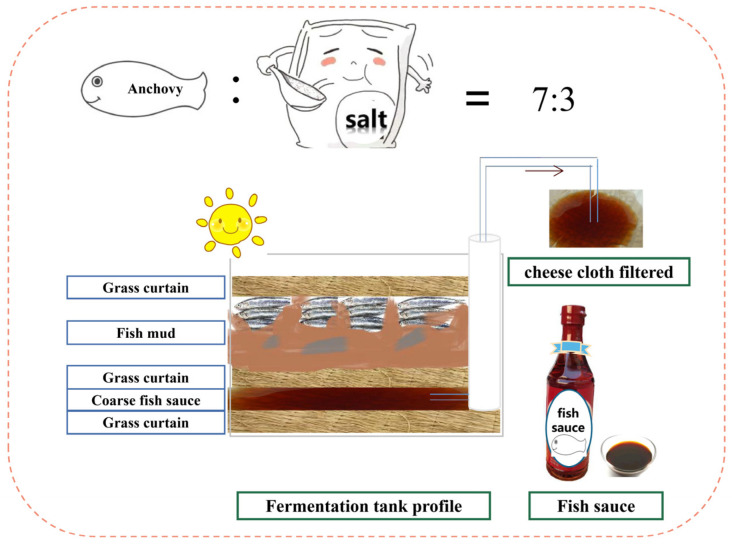
Schematic diagram of fish sauce natural fermentation.

The fish sauce samples were collected from the Rongcheng Baozhiyuan fish sauce factory (Shandong province, PR China). Fish sauce is made by the natural fermentation of anchovies and salt at a ratio of 7:3 for more than 12 months (Figure 1). The annual factory output of the fish sauce is 2000–2500 tons, and it is a large-scale factory in northern fish sauce factories. Samples were collected at 3, 6, 9, 12 and 18 months of fermentation. Three groups of samples were taken in parallel from three fermentation tanks. In order to ensure the uniformity and representativeness of samples, equal amounts (0.25 kg) of samples were taken from the upper, middle and lower parts of each fermentation tank, and the final samples were mixed from the three parts. We placed the sample in the sterile sampling bags and quickly transported them back to the laboratory. Parts of the samples were immediately used for microbial analysis, and the remaining samples were stored at −80 °C.

#### 2.1.2. Fermentation Experiments

Fish sauce samples fermented for 9 months and shrimp paste, Doujiang and Sufu, purchased at the market, were selected as the raw materials to determine the effect of strains on the accumulation of BAs in different fermented foods. The isolated strain (OD_600_ = 0.4) was inoculated into the above-mentioned fermented foods (50 g) with an inoculum of 5% (*v*/*v*), and the fermentation was continued for 7 days at 37 °C. The control group was inoculated with 5% (*v*/*v*) sterilized water in different fermented foods. All experiments were performed in triplicate.

### 2.2. Microbiological Analyses

#### 2.2.1. Microbial Community Analysis

To further analyze the changes in microbial composition in the fish sauce during fermentation, the fish sauce samples were subjected to high-throughput sequencing analysis. Primer pairs 338F and 806R were used to amplify the hypervariable V3–V4 region of bacterial *16S rRNA* gene. The amplified product was purified using the AxyPrep DNA gel extraction kit (Axygen Biosciences, Union City, CA, USA) according to the manufacturer’s instructions, and was run on the Miseq Illumina platform at Majorbio Bio-Pharm Technology Co., Ltd. (Shanghai, China) [21].

#### 2.2.2. Strains Identification

Luria-Bertani (LB) medium with 15% NaCl, de Man Rogosa and Sharp (MRS) medium with 15% NaCl and Plate Count Agar are the main media used in this study. A total of 90 mL of sterile normal saline (0.85%, *v*/*v*) was added to 10 g fish sauce sample, and the mixture was shaken at 37 °C at 150 rpm for 30 min. The mixtures were serially diluted (10^−1^ to 10^−5^) with the sterile normal saline, and 100 µL of each dilution was plated onto the above medium. The single colony was isolated and purified on the corresponding medium at least 4 times to obtain the pure isolates. For the identification of bacteria, the Gen-EluteTM Kit (Tiangen Biotechnology Co., Ltd., Beijing, China) was used to extract the genomic DNA of the bacteria. The bacterial *16S rDNA* gene was amplified with the universal primer pairs 27F and 1492R. The PCR programswere carried out as follows: pre-denaturation at 94 °C for 5 min, followed by 30 cycles at 94 °C for 30 s, 56 °C for 30 s, 72 °C for 1 min and elongation at 72 °C for 10 min. The amplified fragments were then sent to sequencing (Beijing BGI Huada Biotech Co., Ltd., Beijing, China). The pure isolates were stored at −80 °C in the corresponding liquid culture medium containing 32% (*v*/*v*) glycerin [17].

### 2.3. Biogenic Amines Determination

#### 2.3.1. Determination of BAs

Eight BAs or amine hydrochlorides, namely Tryptamine, β-phenylethylamine, putrescine, cadaverine, histamine, tyramine, spermine and spermidine (Aladdin, Shanghai, China), and derivating agent (dansyl chloride (Meilunbio, Dalian, China)) were purchased from Sangon Biotech (Shanghai, China) and Meilun biotech (Dalian, China).

The BA content in each sample was assessed according to the method developed by Sang et al. [21]. In detail, add 80 μL 2 M NaOH, 120 μL saturated NaHCO_3_, and 800 μL dansyl chloride solution (10 mg/mL in acetone) to 400 μL pretreated sample. The mixtures were incubated in water at 45 °C for 40 min. A total of 550 uL of acetonitrile was added to the mixture to dissolve the residue and then the mixture was centrifuged at 3000× *g* for 5 min at 4 °C. The supernatant was filtered through the 0.22 filter for three times. The quantification of BA was carried out using a Huapu S6000 HPLC unit (ACCHROM, Beijing, China) which consisted of an ACCHROM Tnature C18 (4.6 × 250 mm) column coupled to a quaternary pump and a diode array detector. Two elution solutions are (A) ultrapure water and (B) acetonitrile. A 10 µL sample was injected into the column at a flow rate of 1.0 mL/min. The gradient elution program was carried out as follows: 0–10 min, 45% A; 10–15 min, 35–45% A; 15–20 min, 20–35% A; 20–25 min, 20% A; 25–30 min, 10–20% A; 30–33 min, 10% A; 33–35 min, 10–45% A; 35–40 min, 45% A. During analysis, the column temperature was lower than 35 °C, and the absorbance was monitored with a UV detector at 254 nm. Each BA was quantified with a calibration curve generated by analyzing standard BA mixed solution. The experiment was carried out in triplicate.

#### 2.3.2. Pretreatment of Samples of Fish Sauce and Other Fermented Foods

After adding 20 mL of 10% trichloroacetic acid (TCA) (SCR, Shanghai, China) to 5 g of sample to extract biogenic amines, the mixture was left at 4 °C for 2 h. The mixture was centrifuged at 8000× *g* for 10 min at 4 °C. An equal volume of 10% TCA was used to extract the remaining BA in the supernatant again. The obtained mixture was centrifuged at 3000× *g* for 10 min at 4 °C. The BA in the samples was determined according to the method described in Section 2.3.1.

#### 2.3.3. Determination of BA Production and Reduction Properties of Strains

The strains were cultured in a 5 mL medium containing 0.5% (*w*/*v*) histidine, tyrosine, ornithine hydrochloride and lysine (BBI, Shanghai, China). The BA concentrations after 48 h of incubation were determined to evaluate the BA production ability of the strains. The strain was incubated at 37 °C for 12–24 h and then centrifuged at 6000× *g* for 5 min to collect bacterial pellets. The bacterial pellets were washed twice with 0.05 mol/L phosphate buffer (PBS, pH = 7), and then centrifuged at 6000× *g* for 5 min. We added 0.05 mol/L PBS containing putrescine, cadaverine and histamine (100 mg/L) to the bacterial pellet until the OD_600_ of the mixture reached 0.4. The mixture was then cultured at 37 ◦C for 48 h and the residual BAs in the suspension were determined. Sterile PBS (including 100 mg/L putrescine, cadaverine and histamine) was used as a control. The BA reduction efficiency was calculated as:

(1) $$A=\left[\left(C-S\right)÷C\right]\times 100\%$$

*A* is the BA reduction percentage and *C* and *S* are the BA concentrations of the control and strain specimens, respectively [22]. To extract the biogenic amine in the bacterial solution, 9 mL TCA was added to 1 mL bacterial solution, and the mixture was left at 4 °C for 2 h. BAs were determined by the method described in Section 2.3.1.

### 2.4. Statistical Analysis

The statistical software SPSS 26 was used to analyze the significance of the difference by one-way ANOVA method. The high-throughput sequencing analysis was performed using the free online platform of the Majorbio I-Sanger Cloud Platform (www.i-sanger.com, accessed on 2 February 2021). For the data of high-throughput sequencing, raw data were quality-filtered by Fastp (version 0.20.0). Operational taxonomic units (OTUs) were clustered with 97% similarity cutoff using Uparse (version 7.1) (http://www.drive5.com/uparse/, accessed on 2 February 2021). The taxonomy of each 16S rRNA gene sequence was analyzed by RDP Classifier (version 2.2) algorithm (https://sourceforge.net/projects/rdp-classifier/, accessed on 16 July 2021) against the Silva 128/16s_bacteria, using a confidence threshold of 0.7.

### 2.5. Nucleotide Sequence Accession Numbers

The raw reads of bacterial 16S rRNA gene sequencing were deposited into the NCBI Sequence Read Archive (SRA) database (Accession Number: SRR15851749).

## 3. Results

### 3.1. BA Contents in Fish Sauce Samples at Different Fermentation Stages

The content of BA in fish sauce samples at different fermentation stages was shown in Table 1. In the fish sauce samples of five fermentation stages, six main BAs were detected, including histamine (18.39–74.50 mg/kg), tyramine (13.23–20.88 mg/kg), tryptamine (9.59–20.41 mg/kg), β-phenylethylamine (7.63–27.26 mg/kg), putrescine (14.71–60.41 mg/kg), and cadaverine (23.44–90.61 mg/kg), and the content of total BAs ranged from 89.38 to 296.82 mg/kg. Spermidine was not detected in the early period (3–6 months) and was formed during the medium (6–12 months) and late period (12–18 months), while spermine was only detected in the early period of fermentation. During the fermentation process, the concentration of BAs gradually increased. The content of histamine and cadaverine increased to the maximum at the 18th month of fermentation, which was 74.50 mg/kg and 90.61 mg/kg, respectively.

### 3.2. Dynamic Changes of Microorganisms in Fish Sauce Samples during Fermentation

**Figure 2:**
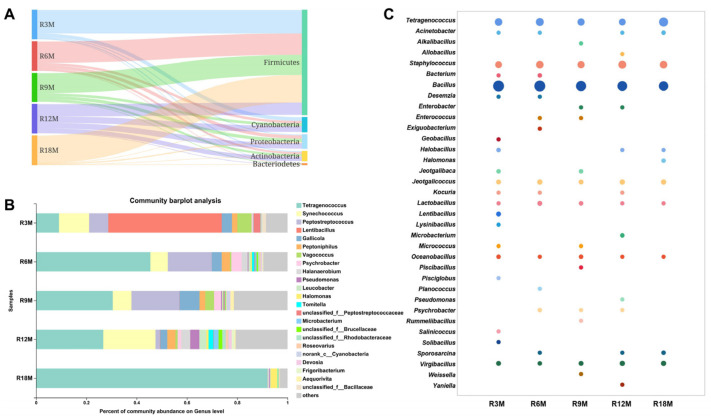
Analysis of bacterial community dynamics in the fish sauce fermentation process. (**A**) Bacterial community dynamics at the phylum level. (**B**) Bacterial community dynamics at the genus level. (**C**) Bacterial community dynamics analysis by traditional separation and screening methods. (R3M, R6M, R9M, R12M, R18M indicated for 3, 6, 9, 12, 18 months, respectively).

We analyzed the change in microbial diversity during fish sauce fermentation by high throughput sequencing. At the phylum level, *Firmicutes* predominated during the entire fermentation, particularly in 18-month samples with up to 94.29% of the total sequences. The species abundance of *Cyanobacteria* was high during the process from 3 months to 12 months of fermentation, especially in 12-month samples (21.10%), while after 18 months of fermentation, the abundance of *Cyanobacteria* dropped sharply to 0.01%. *Proteobacteria* and *Actinobacter* rose rapidly between 3 and 12 months of fermentation, and the abundance reached 19.14% and 15.13%, respectively. The abundance of these two phyla decreased significantly after fermentation to 18 months. (Figure 2A). At the genus level, *Lentibacillus* (approximately 45.90%) was the most dominant bacteria in the early phase of fish sauce fermentation. Then, *Tetragenococcus* (9.15–45.78%) rapidly replaced *Lentibacillus* as the dominant genus at the 6th month of fermentation. In the 18th month of fermentation, *Tetragenococcus* became the most dominant bacteria (approximately 91.99%), and *Halomonas* became the second most dominant genus with an abundance of 2.74%. The genus *Pseudomonas* appeared as the dominant genus during the late fermentation period (9–18 month), especially in the 12-month samples, and the abundance reached 3.69% (Figure 2B).

To further determine the microbial diversity of fish sauce, we used traditional separation and screening methods to isolate and identify the microorganisms in fish sauce. According to *16S rDNA* gene sequence, a total of 284 strains were classified into 35 genera and 77 species (Figure 2C). Some microorganisms contributing to fermentation were isolated, such as *Tetragenococcus*, *Bacillus*, *Staphylococcus*, *Lentibacillus*, *Psychrobacter*, *Halomonas* and *Pseudomonas*. Species level analysis showed that two species of *Tetragenococcus* were found. Among them, the isolation frequency of *Tetragenococcus halophilus* strains was high during the fermentation process. Seven species of *staphylococcus* were also found. *Staphylococcus nepalensis* and *Staphylococcus saprophyticus* were more frequently found in the early period of fermentation, while *Staphylococcus epidermidis, Staphylococcus captis* and *Staphylococcus lentus* were in the later fermentation period. *Lentibacillus* and *Pseudomonas* strains were only isolated in the 3- and 12-month samples, respectively. These results also verified the results of high-throughput sequencing.

**Figure 3:**
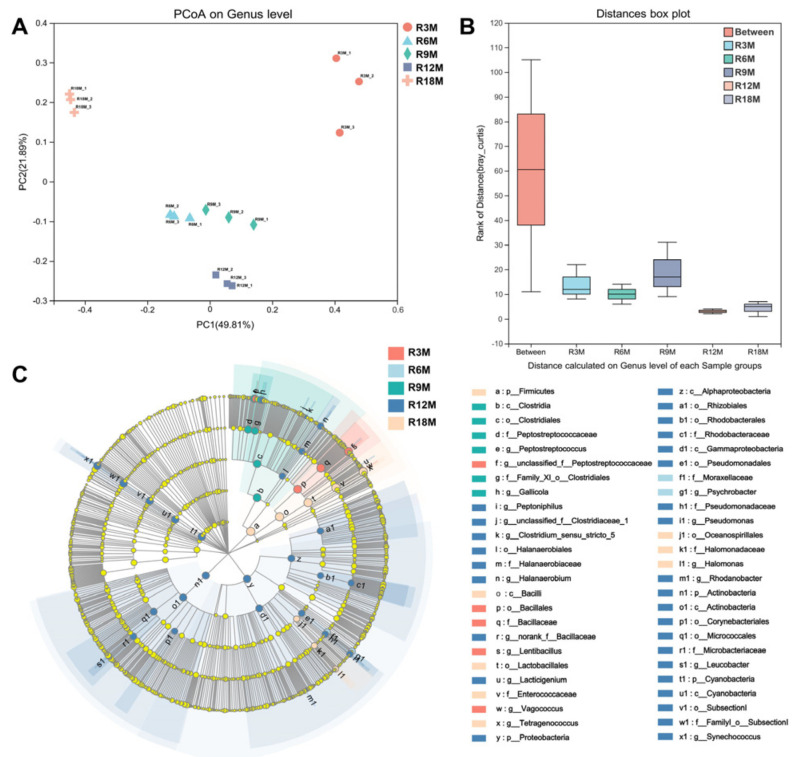
The influence of fermentation time on the microbial composition in fish sauce. (**A**) PCoA (Bray–Curtis). Different samples are represented by points of different colors or shapes. The closer the points are, the more similar the species composition of the samples. (**B**) ANOSIM. The “Between” boxes refer to differences between groups, the others represent differences within each group. (**C**) LEfSe. Microorganisms that are significantly enriched in the corresponding group are represented by nodes with different colors and have a significant impact on the differences between groups. (R3M, R6M, R9M, R12M, R18M indicated for 3, 6, 9, 12, 18 months, respectively).

The effect of fermentation time on the microbial composition of fish sauce was analyzed. Bray–Curtis principal coordinate analysis (PCoA; beta-diversity) was used to qualitatively examine the differences in bacterial composition, and the results showed the clear cluster pattern of the five samples, and the microbiological composition of samples fermented for 6 months was more similar to that at 9 months (Figure 3A). ANOSIM (analysis of similarities) analyzed the significant differences between fish sauce samples in five fermentation stages, indicating that the bacterial composition varied greatly in time distribution (Figure 3B). The communities which had significant differences in the grouping of samples were found by LEfSe analysis. *Lentibacillus*, *Psychrobacter*, *Gallicola*, *Pseudomonas* and *Tetragenococcus* were the representative bacteria in the fish sauce samples fermented for 3, 6, 9, 12 and 18 months, respectively (Figure 3C).

### 3.3. Microbial Contribution to BA Contents in Fish Sauce

**Figure 4:**
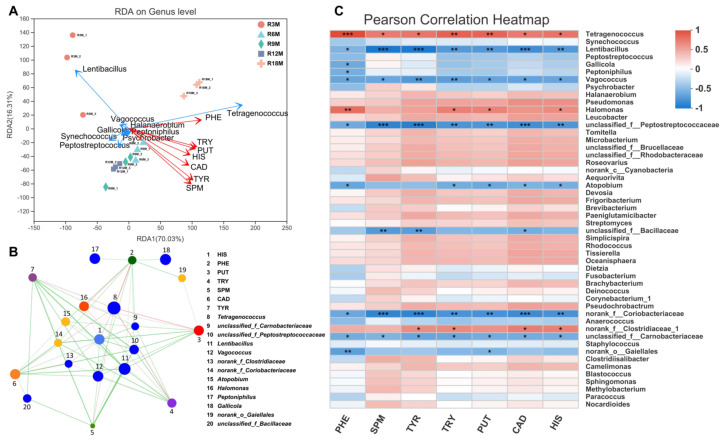
Correlation analysis between microorganisms and BAs during fish sauce fermentation. (**A**) RDA. Different samples are represented by points of different colors or shapes. The length of the arrows represents the degree of impact of environmental factors on the species data. The correlations are represented by the angle between the arrows (acute angle: positive correlation; obtuse angle: negative correlation; right angle: no correlation). (**B**) Correlation network analysis. The size of the nodes indicates the abundance of species, and different colors indicate different species. Red line: positive correlation, green line: negative correlation. (**C**) Pearson correlation heatmap diagram. * 0.01 < *p* ≤ 0.05, ** 0.001 < *p* ≤ 0.01, *** *p* ≤ 0.001. (R3M, R6M, R9M, R12M, R18M indicated for 3, 6, 9, 12, 18 months, respectively. TRY: tryptamine, TYR: tyramine, PHE: β−phenylethylamine, HIS: histamine, PUT: putrescine, CAD: cadaverine, SPM: spermine).

The RDA (redundancy analysis) analysis among BAs, fermentation time and dominant microorganisms are shown in Figure 4A. This result showed that all BAs are positively correlated with fermentation time, especially β-phenylethylamine. Among the dominant microorganisms, *Tetragenococcus* and *Lentibacillus* had the strongest correlation with BAs. *Tetragenococcus* is positively correlated with BAs, while *Lentibacillus* is negatively correlated with BAs. The correlation network analysis between species and BAs shows that most genera were negatively related to BAs. However, some bacterial genera still exhibited as being positively related to BAs (Figure 4B). For example, *Tetragenococcus* is positively correlated with all BAs, *Halomonas* is positively correlated with histamine, β-phenylethylamine, putrescine and tryptamine, and *unidentified genus from family Clostridiaceae* is positively correlated with tyramine, cadaverine, tryptamine and histamine. Pearson correlation analysis showed the correlation between BAs and the top 50 genera. Except for *Tetragenococcus* and *Lentibacillus,* which are strongly correlated with BAs, we found that the dominant genus *Pseudomonas* was weakly positively correlated with BAs, while *Staphylococcus* and *Psychrobacter* were weakly negatively correlated with BAs (Figure 4C).

In order to better illustrate the role of dominant bacteria on the accumulation of BAs, we evaluated the ability of producing the BAs of 100 dominant strains isolated from fish sauce (Table 2). A total of 56.00% of the dominant strains can produce a large amount of BAs. About 65.96% *Tetragenococcus* strains, 45.83% *Staphylococcus* strains, 33.33% *Lentibacilluis* strains and all strains of *Psychrobacter* and *Pseudomonas* had a high ability to produce BAs. These strains may be the main factor causing the large accumulation of BAs in fish sauce. The analysis of the correlation between dominant bacteria with BAs and the determination of BA production ability of the dominant isolates showed that *Tetragenococcus*, *Lentibacillus*, *Psychrobacter*, *Pseudomonas* and *Staphylococcus* and other bacteria had a regulatory effect on the accumulation of BAs in fish sauce fermentation. For example, *Tetragenococcus* positively regulated BAs and promoted the accumulation of BAs, while *Lentibacillus* negatively regulated BAs and inhibited the accumulation of BAs.

### 3.4. Screening of Novel Starter Cultures for Reducing Biogenic Amines

Due to the regulation of microbes on the accumulation of BAs, it is feasible to select microorganisms to reduce the content of BAs in fish sauce. Therefore, eight strains with the lowest BA production capacity (especially histamine) were screened from the genera and their BA reduction ability was determined (Table 3 and Table S1). Staphylococcus strains, particularly *S. nepalensis*, had the best ability to reduce BAs. The reduction efficiencies of *S. nepalensis* 5-5 on putrescine, cadaverine and histamine were significantly higher than other strains, which were 17.64%, 19.80% and 16.77%, respectively. The isolated Lentibacillus strains also showed a high ability to reduce BAs. The ability to reduce the histamine of Lentibacillus salicamp SF-20 was higher than that of Lentibacillus amyloliquefaciens LAM0015. Tetragenococcus halophilus NBRC 12172 and Tetragenococcus muriaticus LMG 18498 had a low ability to reduce BAs, especially histamine. Strains with high BA reduction ability can be used as candidates for reducing BAs in fermented foods.

**Figure 5:**
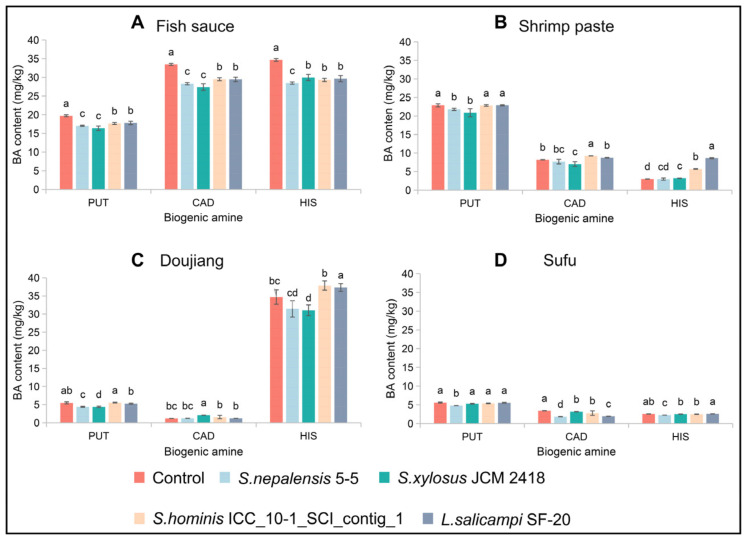
The content of BAs in fermented foods inoculated with different strains (**A**: Fish sauce. **B**: Shrimp paste. **C**: Doujiang. **D**: Sufu.). Different letters (a, b, c, etc.) indicate the mean value of significant difference at *p* < 0.05. (PUT: Putrescine; CAD: Cadaverine; HIS: Histamine).

*S. nepalensis* 5-5, *S. xylosus* JCM 2418, *S. hominis* ICC_10-1_SCI_contig_1 and *L. salicampi* SF-20 might be potential candidates for controlling the BAs due to their high BA reduction ability as well as low BA production activity. In order to ascertain the BA reduction ability of the strains in the food matrix, we inoculated the strains into different fermented foods and determined the BA content in the samples after 7 days of continuous fermentation. All four strains can reduce the BAs in fish sauce, and *S. nepalensis* 5-5 and *S. xylosus* JCM 2418 especially showed the best BA reduction ability. In the shrimp paste, only *S. nepalensis* 5-5 and *S. xylosus* JCM 2418 can reduce the BAs, while the remaining strains increase the BA content. *S. xylosus* JCM 2418 has the highest reducing ability on HIS in Doujiang, while *S. hominis* ICC_10-1_SCI_contig_1 and *L. salicampi* SF-20 increased the content of HIS in Doujiang. *S. nepalensis* 5-5 had the best BA reduction ability in Sufu among all the strains (Figure 5). The above results indicate that *S. nepalensis* 5-5 and *S. xylosus* JCM 2418 had excellent capacities for controlling BAs in fermented food.

## 4. Discussion

Excessive consumption of BAs can cause a variety of harmful effects [11]. Natural fermentation gives the fish sauce a rich microbial community, some of which can produce decarboxylase for the decarboxylation of amino acids to produce BAs, which is detrimental to food safety [2,9]. On the contrary, BAs can also be degraded by the amine oxidase of some bacteria. In this study, six common BAs were detected in the fish sauce samples at five fermentation stages, and putrescine, cadaverine and histamine existed in all samples. The histamine content of the fish sauce sample at the end of fermentation was much higher than the toxic level (50 mg/kg) suggested by the United States FDA [14] (Table 1). BAs could be recognized as signs of the quality and safety of products. Therefore, the contents of BAs in food should be effectively reduced and legitimately controlled.

The microbial community structure changed constantly during the fermentation of fish sauce. This may be due to the introduction of a large number of microorganisms in the natural fermentation mode, which made the microbial composition in the early fermentation samples significantly different from those in other groups. However, as the fermentation progressed, the stability of the microbial composition gradually improved, and the advantages of the fermented genus gradually appeared (Figure 2B and Figure 3A,B). Traditional isolation and screening methods have obtained a large number of *Staphylococcus* strains. The high-throughput sequencing results showed that the abundance of *Staphylococcus* was not very high, but it could be detected at every fermentation stage, indicating that it was also a very important genus for fermentation (Figure 2C).

There are differences in the representative bacterial genera in the samples at different fermentation stages (Figure 3C). *Lentibacillus* is a representative microorganism in fish sauce samples fermented for 3 months, which is considered as a common microorganism in fish sauce [22]. *Psychrobacter* as a genus, often detected in fermented food, was a representative microorganism in fish sauce samples fermented for 6 months. It is reported that the presence of *Psychrobacter celer* can lead to a high production of pleasant volatile aroma compounds such as aldehydes, ketones and sulfur compounds [23]. However, some reports stated that *Psychrobacter* inoculated into cheese might produce HIS [24]. *Pseudomonas* as a representative microorganism in fish sauce samples fermented for 12 months was a common spoilage bacterium, which could produce a large amount of hydrogen sulfide in fish juice and has the ability to produce BAs [25]. *Tetragenococcus* is the dominant genus in each fermentation stage, but the abundance in the samples fermented for 18 months is significantly different from other fermentation stages (the abundance in samples of 3, 6, 9, 12 and 18 months of fermentation was 9.15%, 45.78%, 30.00%, 26.34% and 91.99%, respectively). Therefore, it might be a key genus that determines the quality and safety of the final products. *Tetragenococcus* exhibits certain halotolerant characteristics and plays a major role in sauce flavor [26,27]. In addition, the previous report showed that *Tetragenococcus halophilus* MJ4 clearly repressed the formation of cadaverine during fermentation to improve the safety of fish sauce products [28]. However, it was reported that *Tetragenococcus* had a high BAs production ability, which had a great effect on the accumulation of BAs in sausage, shrimp paste, etc. [17,29]. Some genera detected throughout the fermentation process, such as *Staphylococcus*, also played an important role in the quality and safety of fish sauce products, which is often used as a starter in fermented foods and can be used to control BAs during food fermentation [30].

The microbial composition of fish sauce samples had significant differences in time distribution, and BAs were also related to fermentation time (Figure 4A) [5], indicating that the characteristic microorganisms in samples of specific fermentation time might have a great contribution to the formation of BAs. Therefore, it is necessary to analyze the correlation between the characteristic microorganisms and BAs. The accumulation of BAs in the samples was low at the early fermentation period (3–6 months), and the early representative microorganism, *Lentibacillus* and *Psychrobacter,* were negatively correlated with BAs, suggesting the BA accumulation might be inhibited by *Lentibacillus* and *Psychrobacter* during this period. As the microbial composition of the samples fermented for 6 months was similar to that of 9 months, BA accumulation was also low during the fermentation of fish sauce from 6 to 9 months. *Pseudomonas*, positively related to BAs, was a representative microorganism in fish sauce samples fermented for 12 months. During the fermentation period of 9–12 months, the content of BAs in fish sauce increased greatly, indicating that *Pseudomonas* might be promote the accumulation of BAs. *Tetragenococcus* was dominant during the whole fermentation process, and it was significantly positively correlated with BAs, indicating that *Tetragenococcus* might play a key role in the accumulation of BAs (Figure 4B,C).

To verify the results of the correlation analysis, we tested the BA production capacity of the isolated dominant strains (Table 2). The BA production ability of isolated strains could prove the results of the correlation analysis, indicating that *Tetragenococcus* and *Pseudomonas* may promote the accumulation of BAs, while *Lentibacillus* and *Staphylococcus* may inhibit the accumulation of BAs. Isolated *Psychrobacter* strains produced high BAs, while the results of high-throughput sequencing showed a negative correlation between *Psychrobacter* and BAs, possibly due to the limitations of isolation and screening methods, and the ability to produce BAs was strain specific. The accumulation of BAs in fish sauce fermentation could be affected by changing the species and abundance of dominant microorganisms. The method of adjusting the microbial community structure by changing the fermentation process is very common. The difference of salt concentration in fermentation affected the composition of microorganisms in the samples, and the content of BAs in the products with different salt concentrations was obviously different [16]. In addition, temperature can also affect the accumulation of BAs during fermentation. Delgado-Ospina et al. [31] demonstrated that temperature helped to simulate the off and on of the amine metabolism during cocoa fermentation, thus regulating BA accumulation and reduction. Recently, microbial fermentation as a means to improve the safety and quality of fermented food has been widely used. Xia et al. [32] used *Lactobacillus plantarum* and *Staphylococcus xylosus* as starters to co-inoculate Chinese rice wine. Co-inoculation induced a significant reduction in total BAs (43.7%), putrescine (43.0%), tyramine (42.8%), and histamine (42.6%) content. Therefore, it is feasible to select suitable starters to reduce the accumulation of biogenic amines during the fermentation of fish sauce.

About 44.00% of the dominant strains in this study produce very little Bas; therefore, they may have BA reduction ability and potential as fish sauce starter cultures. Eight isolated strains were selected from the dominant species of fish sauce samples, including four *Staphylococcus* strains, two *Lentibacillus* strains and two *Tetragenococcus* strains, and their BA reduction abilities were evaluated (Table 3). Among them, the *Staphylococcus* and *Lentibacillus* isolates showed high BA reduction ability. The results show that microbial-based solutions can be designed to reduce the BA content in fermented foods [30]. In previous studies, *Staphylococcus* is often used as a starter to reduce BAs in fermented foods. For example, Muhammad et al. [33] found that HIS concentration was reduced by 27.7% by *Staphylococcus carnosus* FS19 and could also reduce other amines during fermentation. Li et al.’s [30] study showed that the *Staphylococcus* isolates including *Staphylococcus pasteuri*, *Staphylococcus epidermidis*, *Staphylococcus carnosus*, and *Staphylococcus simulans* could significantly reduce BAs and have the potential to control BAs in fermented meat products. *Lentibacillus* is a common genus of bacteria in fish sauce [34]. Thus, the effects of Lentibacillus and Staphylococcus isolates on BA accumulation were assessed in different fermented foods to screen the potential starters for BA control (Figure 5). The content of BAs in fermented foods inoculated with *S. nepalensis* 5-5 and *S. xylosus* JCM 2418 were significantly reduced, indicating that the two strains could be utilized as potential candidates for controlling BAs in fermented foods.

## 5. Conclusions

In summary, the bacterial community structure and BA profiles in fish sauce at different fermentation stages were comprehensively evaluated. The dominant bacteria of fish sauce were found to be *Tetragenococcus*, *Lentibacillus*, *Pseudomonas*, *Halomonas*, *Vagococcus* and *Staphylococcus* by high-throughput sequencing. The correlation analysis between microorganisms and BAs indicated that *Tetragenococcus*, *Halomonas* and *Pseudomonas* were positively correlated with BAs, while *Lentibacillus*, *Vagococcus* and *Staphylococcus* were negatively correlated with BAs. By analyzing the BA production capacity of dominant isolates, the accumulation of BAs in fish sauce might be promoted by *Tetragenococcus* and *Halomonas*. Additionally, *S. nepalensis* 5-5 and *S. xylosus* JCM 2418 isolated from fish sauce were applied as starters to fish sauce and other fermented foods for reducing health risk. Our research assessed the BA reducing application of *S. nepalensis* 5-5 and *S. xylosus* JCM 2418 during fish sauce fermentation for the first time, and provided a theory to support the standardized and secure production of fish sauce.

## Figures and Tables

**Table 1:** Content of BAs in fish sauce samples at different fermentation stages.

BAs (mg/kg)	R3M	R6M	R9M	R12M	R18M
TRY	9.59 ± 0.40 ^e^	10.78 ± 0.24 ^d^	12.77 ± 0.34 ^c^	17.76 ± 0.26 ^b^	20.41 ± 0.42 ^a^
PHE	7.63 ± 0.25 ^e^	9.14 ± 0.12 ^d^	13.56 ± 0.62 ^c^	21.24 ± 0.25 ^b^	27.26 ± 0.06 ^a^
PUT	14.71 ± 0.92 ^e^	19.77 ± 0.10 ^d^	28.15 ± 1.25 ^c^	47.67 ± 0.42 ^b^	60.41 ± 0.27 ^a^
CAD	23.44 ± 1.21 ^e^	29.75 ± 0.20 ^d^	42.08 ± 1.90 ^c^	71.03 ± 0.55 ^b^	90.61 ± 0.28 ^a^
HIS	18.39 ± 0.91 ^e^	22.99 ± 0.17 ^d^	32.75 ± 1.48 ^c^	55.59 ± 0.45 ^b^	74.5 ± 0.17 ^a^
TYR	13.23 ± 0.50 ^e^	15.18 ± 0.28 ^d^	17.86 ± 0.59 ^c^	19.07 ± 0.16 ^b^	20.88 ± 0.06 ^a^
SPD	ND	ND	2.41 ± 0.01 ^c^	2.7 ± 0.01 ^b^	2.75 ± 0.002 ^a^
SPM	2.39 ± 0.04 ^a^	2.41 ± 0.01 ^a^	1.98 ± 0.04 ^b^	ND	ND
Total	89.38 ± 3.97 ^e^	110.01 ± 1.01 ^d^	151.55 ± 7.24 ^c^	235.05 ± 1.79 ^b^	296.82 ± 0.71 ^a^

Data are expressed as mean ± SDs (*n* = 3).; ND: not detected. Different letters (a, b, c, d, e) indicate the mean value of significant difference at *p* < 0.05. (R3M, R6M, R9M, R12M, R18M indicated for 3, 6, 9, 12, 18 months, respectively. TRY: tryptamine, TYR: tyramine, PHE: β-phenylethylamine, HIS: histamine, PUT: putrescine, CAD: cadaverine, SPM: spermine, SPD: spermidine).

**Table 2:** Percentage of strains with BA production activities, shown in vitro by the different bacteria species isolated from fermented fish sauce.

Genus	Number of Total Strains	The Percent of BA-Production Strains
*Tetragenococcus*	47 (16)	65.96%
*Staphylococcus*	48 (26)	45.83%
*Lentibacillus*	3 (2)	33.33%
*Psychrobacter*	1 (0)	100.00%
*Pseudomonas*	1 (0)	100.00%

For each genus, the number of isolates with low production of BAs is shown in brackets.

**Table 3:** Percentage of strains with BA reduction activities.

Strains	PUT (%)	CAD (%)	HIS (%)
*Staphylococcus nepalensis* 5-5	17.64 ± 0.44 ^a^	19.80 ± 0.93 ^a^	16.77 ± 1.04 ^a^
*Staphylococcus xylosus* JCM 2418	11.96 ± 0.71 ^b^	6.74 ± 0.31 ^c^	5.04 ± 0.27 ^d^
*Staphylococcus hominis* ICC_10-1_SCI_contig_1	9.32 ± 0.34 ^c^	8.68 ± 0.69 ^b^	7.81 ± 0.73 ^b^
*Staphylococcus capitis* +Y36	9.78 ± 0.37 ^c^	7.26 ± 0.51 ^c^	5.64 ± 0.79 ^cd^
*Lentibacillus salicamp* SF-20	8.62 ± 0.12 ^d^	8.21 ± 0.62 ^bc^	6.87 ± 0.47 ^c^
*Lentibacillus amyloliquefaciens* LAM0015	8.78 ± 0.89 ^cd^	5.26 ± 0.68 ^d^	4.54 ± 0.65 ^d^
*Tetragenococcus halophilus* NBRC 12172	5.34 ± 0.64 ^e^	3.50 ± 0.61 ^e^	2.59 ± 0.63 ^e^
*Tetragenococcus muriaticus* LMG 18498	4.68 ± 0.78 ^e^	3.91 ± 0.24 ^e^	2.00 ± 0.15 ^e^

Data are expressed as mean ± SDs (*n* = 3).; Different letters (a, b, c, d, e.) indicate the mean value of significant difference at *p* < 0.05. (PUT: putrescine; CAD: cadaverine; HIS: histamine)

## Data Availability

The datasets generated for this study are available on request to the corresponding author.

## Supplementary Materials

The following are available online at https://www.mdpi.com/article/10.3390/foods10112572/s1, Table S1: Determination of BA production capacity of dominant isolates.
