# Supplementary material for: Contribution of Microorganisms to Biogenic Amine Accumulation during Fish Sauce Fermentation and Screening of Novel Starters
Source: Foods. 2021 Oct 25;10(11):2572. doi: 10.3390/foods10112572 (PMC8621993; doi:10.3390/foods10112572)
Supplement: Supplementary file 1 [file foods-10-02572-s001.zip › foods-1403772-supplementary.pdf]

Table S1 Determination of BA production capacity of dominant isolates.

Strain	Accession number	PUT	CAD	HIS	TYR
S.saprophyticus FDAARGOS_336	CP022056.2	1.53±0.16	3.11±0.33	4.54±0.44	8.58±1.80
S.saprophyticus 201707CJKOP-63	MG309499.1	5.16±0.43	5.90±1.58	5.35±0.43	9.10±0.82
T.halophilus MJ4	CP012047.1	5.79±1.40	3.99±0.81	5.48±0.99	5.10±0.19
S.nepalensis 5-5	MH491965.1	1.88±0.43	3.03±0.28	2.92±0.59	7.55±0.60
S.nepalensis 92N2	KX495213.1	2.35±0.97	3.84±0.21	2.01±0.14	10.42±0.23
T.halophilus KUD23	CP020017.1	6.28±0.04	2.68±0.54	3.33±0.27	7.55±0.32
S.saprophyticus BBN4P-01d	FJ357619.1	2.33±0.36	2.81±0.63	2.82±0.76	7.87±0.33
T.sp.HGA-2	MG988273.1	2.17±0.56	3.91±1.06	6.20±0.09	9.74±0.25
T.halophilus NBRC 12172	AP012046.1	2.11±0.62	3.16±0.64	6.64±0.72	10.53±0.34
S.saprophyticus subsp. bovis E7-5c	KT989529.1	ND	13.74±1.01	5.07±0.91	6.28±0.69
T.halophilus LMG 26042	CP027768.1	11.06±0.46	7.86±0.46	8.10±0.73	7.96±0.57
T.sp.HGA-2	MG988273.1	2.22±0.45	3.18±0.17	5.94±0.73	9.50±0.02
S.nepalensis JS11	CP017466.1	1.22±0.09	2.25±0.21	3.59±0.47	8.47±0.12
T.halophilus	AB041346.1	2.24±0.22	2.18±0.30	2.96±0.31	3.45±0.50
T.halophilus	AB041346.1	8.21±0.98	6.40±0.59	7.47±0.53	11.53±0.67
T.halophilus MJ4	CP012047.1	6.40±0.49	6.50±0.08	5.62±0.06	11.26±0.13
T.halophilus MJ4	CP012047.1	1.68±0.42	3.31±0.30	5.75±0.50	9.89±1.24
T.halophilus	AB041346.1	9.61±0.86	5.00±0.46	5.82±0.92	8.24±0.07
S.lentus 3-4	MH491955.1	2.77±0.04	3.29±0.02	2.32±0.05	3.53±0.15
T.halophilus GAM-1	MK063724.1	2.07±0.73	2.21±0.20	3.56±0.21	12.47±0.27
S.nepalensis 5-5	MH491965.1	1.49±0.17	4.11±0.42	2.74±0.26	8.48±0.65
S.saprophyticus FDAARGOS_336	CP022056.2	1.13±0.05	2.12±0.38	2.79±0.27	12.51±0.47
S.nepalensis muzW280	KM010144.1	1.66±0.37	2.64±0.35	2.56±0.46	13.03±0.41
T.halophilus NBRC 12172	AP012046.1	2.54±0.09	2.11±0.38	ND	13.19±0.35
L.amyloliquefaciens LAM0015	NR_146041.1	ND	3.92±0.33	ND	10.57±0.17
S.capitis +Y36	JX113249.1	1.96±0.004	3.42±0.07	ND	8.62±1.02
L.salicampi SF-20	NR_025659.1	1.96±0.12	5.65±0.38	ND	ND
S.hominis ICC_10-1_SCI_contig_1	MK574685.1	1.20±0.01	2.61±0.36	ND	ND
T.muriaticus PMC-11-5	KM042034.1	1.32±0.34	1.95±0.22	4.20±0.51	14.18±1.04
S.nepalensis 5-5	MH491965.1	ND	2.20±0.32	2.50±0.06	ND
S.nepalensis 5-5	MH491965.1	ND	ND	ND	4.52±0.26
T.muriaticus LMG 18498	NR_116418.1	2.10±0.26	2.84±0.13	2.17±0.09	ND
S.saprophyticus 201707CJKOP-63	MG309499.1	1.84±0.56	4.64±0.34	2.84±0.68	6.00±0.05
S.nepalensis 5-5	MH491965.1	1.32±0.27	2.60±0.19	2.70±0.55	ND
S.nepalensis 5-5	MH491965.1	1.26±0.11	2.26±0.08	2.52±0.25	ND
S.xylosus JCM 2418	NR_113350.1	ND	ND	ND	10.44±0.57
T.muriaticus	LC306856.1	7.52±0.47	7.11±0.06	4.94±0.16	8.87±0.39
S.saprophyticus 91A (BP3)	KF254615.1	1.43±0.20	2.36±0.55	2.66±0.19	13.47±0.74
S.saprophyticus H72	KU922275.1	1.52±0.39	2.15±0.22	3.28±0.21	11.65±0.74
S.saprophyticus 201707CJKOP-63	MG309499.1	2.48±0.10	3.11±0.24	5.41±0.26	14.07±1.32
S.saprophyticus CC01	MK618636.1	2.07±0.05	3.26±0.19	2.83±0.23	15.52±0.97

S.nepalensis muzW280	KM010144.1	3.05±0.16	ND	3.08±0.64	13.57±0.88
S.saprophyticus TUST017	KC456631.1	2.84±0.19	2.82±0.27	2.27±0.05	13.94±0.92
S.epidermidis CG17	MK618611.1	1.08±0.04	2.64±0.42	2.13±0.49	2.54±0.49
S.epidermidis HBUM07083	MF662509.1	552.41±6.42	111.56±0.38	2.87±0.03	2.60±0.19
S.epidermidis PM221	HG813242.1	496.92±24.14	99.60±4.62	2.39±0.08	3.01±0.52
S.epidermidis ATCC 12228	AE015929.1	541.76±11.20	118.21±1.99	1.92±0.04	2.91±0.08
S.nepalensis JS11	CP017466.1	395.13±14.63	9.18±0.46	2.61±0.03	2.45±0.01
S.nepalensis JS11	CP017466.1	314.27±39.27	10.05±1.01	2.21±0.29	2.24±0.13
S.nepalensis RY31-2	AB697721.1	493.65±34.81	33.20±2.38	1.84±0.14	2.73±0.30
S.nepalensis JS11	CP017466.1	576.26±6.62	188.12±2.41	2.10±0.30	52.25±0.42
T.halophilus FSB201	AB041346.1	481.83±67.23	201.19±28.21	1.99±0.26	2.97±0.85
S.epidermidis CG17	MK618611.1	540.70±8.76	249.88±4.23	2.41±0.48	43.98±0.92
S.nepalensis muzW280	KM010144.1	253.36±15.14	5.58±0.13	2.51±0.27	3.07±0.16
S.epidermidis st9	MF102140.1	171.41±10.74	3.55±0.15	2.73±0.12	2.62±0.32
S.warneri PDS_PXHF51	MF043130.1	241.15±2.51	5.52±0.06	2.51±0.07	3.08±0.06
S.epidermidis BMC2N11_1	MH050411.1	564.90±15.20	199.25±5.68	2.17±0.16	2.63±0.04
T.sp. JNURIC-D12	GQ150506.1	504.61±56.16	178.77±20.33	2.76±0.42	2.16±0.26
S.capitis 3P20	MF033474.1	489.13±58.79	47.51±5.38	1.94±0.09	2.83±0.31
S.hominis BMC3N13_2_1	MG996860.1	555.03±28.45	68.62±3.74	2.97±0.29	2.64±0.11
S.epidermidis 3-13	MK880646.1	532.74±2.54	57.86±0.53	1.94±0.02	2.41±0.07
S.epidermidis CAU8047	MF429628.1	559.51±15.88	222.90±6.37	2.40±0.47	58.52±1.66
S.nepalensis JS9	CP017459.1	559.64±7.20	183.58±2.61	2.16±0.09	52.30±0.16
S.saprophyticus CC01	MK618636.1	529.01±12.04	42.53±0.83	1.88±0.02	2.54±0.13
S.epidermidis HCD27-5	MH111598.1	216.08±0.58	4.63±0.03	2.30±0.06	ND
S.epidermidis P32_BA1H	MK883070.1	158.49±1.91	3.10±0.08	2.12±0.12	2.55±0.08
S.nepalensis CW1	NR_028996.1	245.26±13.19	5.86±0.29	2.19±0.11	3.08±0.18
S.nepalensis CW1	NR_028996.1	302.84±15.77	5.38±0.15	2.82±0.05	2.79±0.07
S.nepalensis CW1	NR_028996.1	396.94±3.50	6.37±0.64	ND	ND
T.halophilus MRS-1	MK063722.1	555.47±17.48	128.31±4.46	3.07±0.23	1.72±0.02
T.halophilus subsp. GZH2-18	MG654641.1	564.80±20.54	162.68±6.10	2.65±0.28	2.77±0.08
T.halophilus MRS-1	MK063722.1	231.04±5.71	5.49±0.33	2.42±0.40	3.73±0.98
T.halophilus FSB201	AB041346.1	549.60±15.62	55.87±1.39	2.24±0.03	2.77±0.07
T.halophilus NBRC 12172	AP012046.1	555.86±10.47	189.35±3.33	2.37±0.41	59.09±0.95
T.halophilus GAM-1	MK063724.1	372.52±0.63	7.97±0.01	2.62±0.05	ND
T.halophilus FSB201	AB041346.1	548.93±17.95	180.58±5.69	2.13±0.09	51.77±2.37
T.sp.SZ-B-2	KC170304.1	603.73±34.41	213.56±11.89	2.79±0.17	ND
T.halophilus NBRC 12172	AP012046.1	546.49±29.16	101.23±5.49	2.03±0.22	2.78±0.15
T.halophilus MRS-1	MK063722.1	164.77±3.56	3.30±0.19	1.97±0.09	2.59±0.09
T.halophilus MJ4	CP012047.1	402.29±6.52	6.15±0.10	ND	ND
T.halophilus GAM-1	MK063724.1	554.29±48.94	68.32±6.19	2.36±0.24	2.28±0.12
T.muriaticus JCM 10007	LC306856.1	565.92±10.39	113.66±1.90	2.08±0.02	ND
T.muriaticus 15-6	LC136876.1	509.23±11.95	368.07±7.12	2.45±0.24	2.72±0.33
T.muriaticus LMG 18498	NR_116418.1	197.82±2.94	7.29±0.69	ND	3.02±0.02

T.muriaticus 10-1	AB822564.1	147.80±5.40	2.74±0.03	2.29±0.04	2.59±0.14
T.sp.SZ-B-2	KC170304.1	514.74±20.48	118.94±4.87	2.81±0.20	ND
T.halophilus FSB201	AB041346.1	562.93±9.16	183.81±2.37	1.77±0.05	52.62±0.59
T.halophilus FSB201	AB041346.1	552.61±13.09	180.46±4.42	2.08±0.10	50.07±1.11
T.sp.SZ-B-2	KC170304.1	569.45±8.58	114.15±1.33	2.49±0.08	2.91±0.05
T.halophilus GAM-1	MK063724.1	517.96±17.68	57.70±1.77	2.04±0.03	2.31±0.07
T.muriaticus 15-6	LC136876.1	586.31±9.39	199.35±2.29	2.65±0.45	62.51±0.90
T.muriaticus 15-6	LC136876.1	377.28±34.62	8.79±0.41	2.56±0.13	2.39±0.09
T.muriaticus 15-6	LC136876.1	527.96±27.24	232.44±12.13	2.53±0.05	3.20±0.16
T.muriaticus JCM 10007	LC306856.1	410.30±15.09	9.39±0.33	2.35±0.32	2.38±0.05
T.muriaticus JCM 10007	LC306856.1	568.96±4.50	251.17±2.26	2.67±0.12	3.10±0.23
T.halophilus KUD23	CP020017.1	576.83±11.21	187.76±3.68	2.09±0.04	51.87±0.78
T.muriaticus JCM 10007	LC306856.1	581.46±22.05	117.41±3.79	2.69±0.13	2.90±0.12
Psychrobacter MRYB_s	MRYB01000024	558.16±22.46	62.78±2.70	2.36±0.16	2.57±0.18
Pseudomonas stutzeri	CP002881	409.16±21.10	9.47±0.51	3.10±0.72	2.49±0.17
L.salicampi SF-20	NR_025659.1	580.29±5.79	197.78±1.61	2.60±0.18	61.52±0.22

Data are expressed as mean ± SDs (n=3).; ND: not detected. Different letters (a, b, c, etc.) indicate the mean value of significant difference at $P<0.05$. (R3M, R6M, R9M, R12M, R18M indicated for 3, 6, 9,12,18 months, respectively. PUT: putrescine, CAD: cadaverine, HIS: histamine, TYR: tyramine).
